# Cluster Analysis of Urban Acoustic Environments on Barcelona Sensor Network Data

**DOI:** 10.3390/ijerph18168271

**Published:** 2021-08-04

**Authors:** Antonio Pita, Francisco J. Rodriguez, Juan M. Navarro

**Affiliations:** Research Group in Advanced Telecommunications (GRITA), UCAM Universidad Católica de Murcia, 30107 Guadalupe, Spain; apita@alu.ucam.edu (A.P.); frodriguez@ucam.edu (F.J.R.)

**Keywords:** environmental noise assessment, clustering, k-means, strategic noise map, urban acoustic environment, wireless sensor network data

## Abstract

As cities grow in size and number of inhabitants, continuous monitoring of the environmental impact of sound sources becomes essential for the assessment of the urban acoustic environments. This requires the use of management systems that should be fed with large amounts of data captured by acoustic sensors, mostly remote nodes that belong to a wireless acoustic sensor network. These systems help city managers to conduct data-driven analysis and propose action plans in different areas of the city, for instance, to reduce citizens’ exposure to noise. In this paper, unsupervised learning techniques are applied to discover different behavior patterns, both time and space, of sound pressure levels captured by acoustic sensors and to cluster them allowing the identification of various urban acoustic environments. In this approach, the categorization of urban acoustic environments is based on a clustering algorithm using yearly acoustic indexes, such as $L_{\mathrm{day}}$, $L_{\mathrm{evening}}$, $L_{\mathrm{night}}$ and standard deviation of $L_{\mathrm{den}}$. Data collected over three years by a network of acoustic sensors deployed in the city of Barcelona, Spain, are used to train several clustering methods. Comparison between methods concludes that the k-means algorithm has the best performance for these data. After an analysis of several solutions, an optimal clustering of four groups of nodes is chosen. Geographical analysis of the clusters shows insights about the relation between nodes and areas of the city, detecting clusters that are close to urban roads, residential areas and leisure areas mostly. Moreover, temporal analysis of the clusters gives information about their stability. Using one-year size of the sliding window, changes in the membership of nodes in the clusters regarding tendency of the acoustic environments are discovered. In contrast, using one-month windowing, changes due to seasonality and special events, such as COVID-19 lockdown, are recognized. Finally, the sensor clusters obtained by the algorithm are compared with the areas defined in the strategic noise map, previously created by the Barcelona city council. The developed k-means model identified most of the locations found on the overcoming map and also discovered a new area.

## 1. Introduction

As the size of cities grows, the well-being and quality of life of citizens have become a priority for city managers [1]. Although it is well known that noise is one of the pollutants of greatest concern to citizens [2] and the World Health Organization has recently recommended the reduction of exposure to noise from the most common sources of community noise [3], other factors of the acoustic environment in addition to excessive noise levels should be taken into account in its assessment.

The European directive 2002/49/EC [4] encouraged agglomerations of people, i.e., cities or groups of cities nearby, to create their strategic noise mapping (SNM) sharing the results with citizens. Moreover, the results of these noise maps led to the establishment of noise-reduction action plans where noise exposure protection zones are defined. To create performance reports with the data obtained in the strategic noise map and to define special noise protection areas within the city, data are usually analyzed by descriptive analysis, with basic statistics such as the average or median of the defined noise indicator obtained for the overall assessment period. In general, using these statistics, two main types of areas are proposed relying on the places where values are higher than a certain recommended sound level, known as special regime areas, and others where their noise exposure is lower than the average, known as quiet areas.

In recent years, large cities are deploying Wireless Acoustic Sensor Networks (WASN), based on Internet of Things (IoT) technologies [5], in order to perform continuous monitoring of environmental acoustic parameters at many locations [6]. The acoustic nodes that compose these networks continuously capture information about the sound environment over long periods of time, generating a large amount of data. These acoustic data, together with further environmental data, such as water quality [7] or air pollution [8], are being used by city managers to make decisions and propose improvement actions. Moreover, this smart city system has given rise to the creation of the so-called dynamic noise maps where SNM are more often updated, each day for instance, by integrating data obtained from acoustic sensors and the application of predictive models of sound propagation in cities [9].

The advantages provided by IoT technology [10], including low power consumption of the equipment and wide area coverage, allow for easy deployment of a large number of devices throughout the city as well as transmitting values of acoustic parameters every short time interval, e.g., every minute [11]. The analysis of this large amount of information generated by the WASN can be considered a big data problem [12].

Therefore, this work is focused on performing a cluster analysis of urban acoustic environments, evaluating the suitability of applying an unsupervised machine learning model to automatically classify several groups of nodes with different behavior patterns, both time and space, of sound pressure levels. For the description of this technique, data captured during three years in a WASN deployed in the city of Barcelona, Spain, are used [13]. In this work, the categorization of urban acoustic environments is based on a clustering algorithm using the following yearly acoustic indexes, $L_{\mathrm{day}}$, $L_{\mathrm{evening}}$, $L_{\mathrm{night}}$ and standard deviation of $L_{\mathrm{den}}$. A detailed analysis of the obtained clusters is conducted, showing both geographical and temporal additional information to that provided by the city’s SNM [14].

This paper is organised into the following sections. After this introduction, a review of the state of the art of machine learning in environmental acoustics is presented in Section 2. Section 3 presents the data-set and the proposed methodology for unsupervised identification. In next Section 4, results obtained from the analysis are shown and discussed. Finally, Section 5 provides the main conclusions of this research.

## 2. Machine Learning for Analysis of Environmental Acoustics

Machine learning (ML) is a type of artificial intelligence whereby an algorithm or method will extract patterns out of data [15]. ML methods are often divided into three major categories: supervised, unsupervised and reinforced learning [15]. The second is being used in this work, in which, in contrast to supervised and reinforced learning, no labeled input and output data are needed to train the model. The goal of these unsupervised techniques is to find out interesting or useful structures within the data.

As in other research fields, ML is being applied in the area of acoustics and audio signal processing [16]. The application of ML in acoustics is a field of research that has recently attracted great interest in the scientific community. Application examples can be found in a wide range of acoustics fields, such as speech signal processing [17], underwater acoustics [18], medical diagnosis [19], design of acoustic materials [20], bioacoustics [21], room acoustics [22] and environmental acoustics [23].

Signals generated by sound sources, e.g., human speech and musical instruments, contain useful insights that can be used by ML techniques to detect and model complex patterns. Regarding ML approaches, sound captured by acoustic transducers can be classified into two groups depending on the nature of the data created: (i) audio signal, from which it is possible to apply techniques such as event detection [24], classification of sound sources [25], and source location [26], and (ii) acoustic parameters calculated from the audio signal, that have been used to predict sound pressure level values [27] or estimate loudness level values [28] for instance.

As enunciated above, ML techniques are data-driven and they are typically fed with a large amount of data to obtain optimal results. The acquisition and processing of these data require advanced monitoring and management systems. Technological advances have developed new ways of obtaining massive data on environmental quality parameters in cities, the most commonly used being the crowd-sourced data using smart-phone applications [29] and the deployment of wireless acoustic sensor networks [30]. WASN consists of a set of nodes with acoustic transducers that are deployed at locations in the area of interest. These acoustic nodes continuously capture sound with high quality, allow long-term monitoring of urban acoustic environments [31], and also can contribute to create dynamic noise maps [32].

During the last few years, WASNs have been deployed in cities around the world and several studies have been published regarding machine learning techniques for environmental acoustics. Most of the works found in the literature apply supervised machine learning methods to the audio signal, the first group that was defined above. In this approach, the method is firstly trained with labeled data-set, i.e., annotated sound recordings. After the resulting model is evaluated and optimized, the algorithm is then implemented and run in the acoustic nodes.

In the city of New York, a large data-set [33] of labeled audio recording was created by taking advantage of a WASN [34] for the development and evaluation of machine learning techniques, also known as deep-learning techniques because of the high amount of data used to train the model, for real-world urban noise monitoring. Using this data-set, methods for both detection [35] and classification [36] of acoustic scenes and events have been carried out. Recently, a deep learning structure has been developed with this data-set for sound event retrieval [37] of urban sound events, such as car horns and human speech, on multi-label audio recordings.

In an European project, DYNAMAP [38], several machine learning techniques were evaluated for anomalous noise source detection [39], such as birds, people talking, sirens, etc., in order to remove unrelated to road traffic noise events, and then, generate a noise map.

Other supervised ML techniques were applied for sound source classification. A pattern classification algorithm, using Mel-frequency cepstral coefficients as features, was presented in Reference [40] to identify the main noise source of the acoustic environment. Two types of supervised classifiers, Gaussian mixture model and artificial neural networks, were compared in this latter work. An aggregation scheme that combine local features, short-term sound recording features, with long-term descriptive statistics was presented by Ye et al. [41] using a convolutional neural network for the classification of urban sound events.

On the other hand, the application of machine learning to acoustic parameters calculated from the audio signal is a promising topic wherein there are still a few publications that use their advantages to create analytical models in the environmental acoustics field. Segura-Garcia et al. [42] explored the application of the ordinary Kriging technique to perform spatial interpolation of sound pressure level values obtained by a WASN in a small town and automatically generate a noise map. In Reference [43], predicted road-traffic noise level produced with a noise mapping software together with urban form indicators were considered to develop a neural network model. With this machine learning model, statistical noise maps for other cities can be estimated. Recently, a Long Short-Term Memory deep neural network technique was presented to model temporal dependency of sound levels and therefore to predict near-time future values at a certain location [27].

Other studies in the literature implement machine learning algorithms to create models for predicting sound pressure levels at a location. To do this, instead of using acoustic data as input, they use features of location of the sound source, for instance traffic flow and street width to predict road traffic noise [44,45]. In Reference [46], geospatial features are used as input of a random forest algorithm obtaining a model to predict seasonal sound pressure level at different locations. Neural networks can also be used to estimate the sound pressure level that will be produced by an aerofoil in its design phase [47].

Within the previously cited DYNAMAP project [38], which aim is to develop a dynamic noise mapping system of road traffic noise, unsupervised machine learning techniques including clustering and dimensionality reduction have been used to optimize the choice and the number of monitoring sites [48]. Using hourly averaged $L_{\mathrm{Aeq}1h}$ acoustic data of a 24 h measurement campaign in the city of Milan, Italy, a methodology for a more efficient way to estimate the mean $L_{d}$ and $L_{n}$ levels in urban roads compared with the legislative road classification [9] was presented. Moreover, in order to associate each of the streets of the pilot zone with one of the two noise profiles detected in the clustering and then calculate the dynamic map, different non-acoustic parameters were evaluated [9]. Recently, the intermittency ratio indicator was combined with the $L_{\mathrm{Aeq}1h}$ data to improve the classification of different types of road in two identified clusters [49].

In this current research, an analysis of urban acoustic environments of the city of Barcelona is made applying clustering techniques for the identification and classification of different urban acoustic profiles, rather than only urban roads. To achieve this goal, data collected in a long-term period of three years by a WASN are used to train different clustering techniques. In our approach, the categorization is based on a clustering algorithm using yearly acoustic indexes, instead of daily based, allowing to perform comparison with the special acoustic zones defined in the city’s SNM.

## 3. Materials and Methods

In this section, the acoustic data-set and the statistics calculated are introduced first. Then, a classical descriptive analysis is briefly presented. Finally, the performed unsupervised learning method is described.

### 3.1. Data-Set Definition

**Figure 1:**
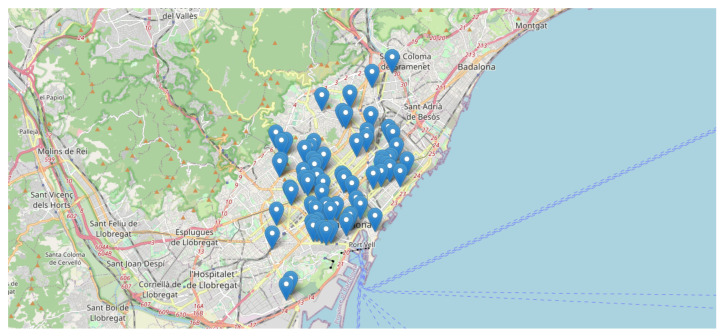
Map showing the location of the 70 acoustic nodes deployed in the city of Barcelona, Spain.

The network of acoustic nodes deployed in Barcelona by the city council during last years consists of 86 sound sensors [13,50]. The data-set used in this research was collected by 70 of the 86 sound sensors which provide long-term analysis, from January 2018 until December 2020. As it is shown in Figure 1, the acoustic nodes are evenly distributed throughout the city, but the city center concentrates the largest number of nodes.

Each node captures sound pressure of its location in a continuous mode, 24 h/7 days a week, using a Cesva TA120 [51] remote sonometer. The accuracy of this type of sensors is defined as class 1 precision sensor according to the International Standard IEC 61672-1 [52]. Most of the sensors are attached to post lamps or similar urban structures at about 4 m above the floor level as is required in ISO1996-2 [53]. Then, the node transmits the dataframe to the central database that stores and processes all the data [13] to be shown in the *Plataforma de Sensors i Actuadors de Barcelona* [54], also known as smart city platform.

The sound pressure p(t) is usually measured continuously over a given time period $T=[t_{1},t_{2}]$ for all t∈T, to quantify the sound level on a single value using the equivalent sound pressure level in dB, denoted as $L_{\mathrm{eq}T}$ [53],

(1) $$L_{\mathrm{eq}T}=10\cdot \log\left[\frac{1}{T}\int_{t_{1}}^{t_{2}}\frac{p^{2}{\left(t\right)}_{}}{p_{0}^{2}}dt\right]\mathrm{where}\;T=t_{2}-t_{1},$$

where $p_{0}$ is the sound pressure reference value equal to 20 μPa. In particular, deployed nodes compute the A frequency-weighting equivalent sound pressure level of one minute period, denoted as $L_{\mathrm{Aeq}1m}$ in dBA unit, applying Equation (1).

In this work, sound pressure level results are presented applying a long-term average of $L_{\mathrm{Aeq}1m}$. Different time periods *T* can be defined, for instance, it is denoted as $L_{\mathrm{Aeq}1d}$ for a 24 h day period and $L_{\mathrm{Aeq}1y}$ for a generic year period. Moreover, the equivalent sound pressure level in a specific year *Y* is denoted as $L_{\mathrm{Aeq}Y}$, for instance $L_{\mathrm{Aeq}2020}$ represents the equivalent sound pressure level for 2020. These values are calculated using an energetic average with the following Equation [53],

(2) $$L_{\mathrm{Aeq}T}=10\cdot \log\left[\frac{1}{n}\sum_{i=1}^{n}10^{\frac{L_{{\mathrm{Aeq}}_{i}}}{10}}\right],$$

where *n* is the total number of 1-unit time intervals in period *T* and $L_{{\mathrm{Aeq}}_{i}}$ is the equivalent sound pressure level in the interval *i* obtained by the sensor applying Equation (1). For instance, to calculate $L_{\mathrm{Aeq}1h}$, 60 values of $L_{\mathrm{Aeq}1m}$ are averaged.

The data provided by the Barcelona city council contains acquired data from January 2018 until December 2020, exported from the smart city platform in several Excel^™^ files in a semicolon tabulated format with a total storage size of 488 MBytes. After the data is prepared, see Section 3.2 for details, several acoustics indicators are calculated regarding Directive 2002/49/EC [4] in order to perform a descriptive statistical analysis, also discussed in Section 3.2, and to calculate the clustering model, presented in Section 3.3. This Directive [4] establishes that member states must calculate the acoustic parameters $L_{\mathrm{den}}$ and $L_{\mathrm{night}}$ for the preparation and revision of the SNM. $L_{\mathrm{den}}$, defined in Equation (3), refers to the day–evening–night noise indicator obtained for an overall assessment period, which is usually a one-year period.

(3) $$L_{\mathrm{den}}=10\cdot \log\left[\frac{1}{24}\left(12\cdot 10^{\frac{L_{\mathrm{day}}}{10}}+4\cdot 10^{\frac{L_{\mathrm{evening}}+5}{10}}+8\cdot 10^{\frac{L_{\mathrm{night}}+10}{10}}\right)\right],$$

where $L_{\mathrm{day}}$, $L_{\mathrm{evening}}$ and $L_{\mathrm{night}}$, also denoted as $L_{d}$, $L_{e}$ and $L_{n}$, respectively, are the A-weighted long-term average sound level. In this paper, $L_{d}$, $L_{e}$ and $L_{n}$ are calculated using Equation (2), determined over all the day periods (07:00–19:00), evening periods (19:00–23:00) and night periods (23:00–07:00), respectively, over the assessment period.

### 3.2. Data Preparation and Exploratory Data Analysis

Previous to the application of the machine learning technique, the raw data in Excel^™^ files has to be prepared in a format that enables analysis and model design. This preparation phase includes data cleaning and feature selection.

Firstly, a data quality analysis was conducted, identifying nulls and the completeness of the data. Due to some technical mistakes, such as connections errors, maintenance and breaks, all the information is not usually available. Therefore, an analysis of the completeness of the data must be carried out to identify the amount of available and missing data. This analysis resulted in a total data-set with 97,181,718 records, i.e., 97,181,718 min, equivalent to 1,619,695 h, 67,487 days or 184 years. A total of 1,735,999 of the records were nulls (1.76%). Some values of this completeness analysis for several nodes are shown in Table 1 as an example. This table includes information regarding the first day with available data, the amount of days with records and the amount of minutes with valid or null records.

As Table 1 shows, different amount of records are available for each node. The main reason is that the nodes were deployed on different dates. In fact, there are nodes that were deployed in early 2019.

Secondly, using this prepared data some statistics were calculated to perform the analysis. These statistics are usually called Key Performance Indicators (KPIs) when they are applied in data-driven decision making. The statistics obtained in this research were processed in a daily and yearly assessment period. To calculate the daily statistics, only the non-null values were considered. Regarding the yearly statistics, the days without data were removed.

Together with $L_{d}$, $L_{e}$, $L_{n}$ and $L_{\mathrm{den}}$, percentile values were also estimated. $P_{N}$ denotes percentile values below which *N*% of the observations may be found. The reader should note that in acoustics, the literature defines $L_{N}$ as the level exceeded for *N*% of the time [53], thus for instance $P_{90}$ corresponds with $L_{10}$. In Table 2, calculated daily statistics for node $BCN_{1}$ in a 15 days period are shown as an example.

**Figure 2:**
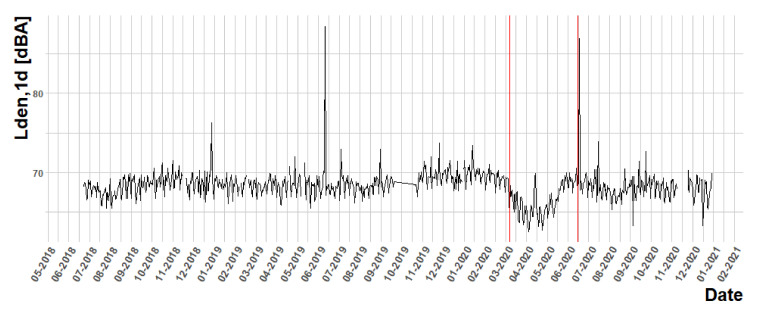
$L_{\mathrm{den}1d}$ time series for node $BCN_{1}$. Note that Spanish lockdown corresponds with the period between red lines.

Once the KPIs are obtained, some basic exploratory analysis can be performed to look for some important features of the data. Although this is not the main objective of this paper, some results are shown to illustrate the experiment. For instance, the sound pressure level time series can be analyzed for each node independently. Figure 2 shows the $L_{\mathrm{den}1d}$ statistics along the available dates. The red vertical lines delimit the period of national state of alarm decreed by the country, with a lockdown from 15 March 2020 to 21 June 2020. Through these graphs, a discussion could arise regarding the effects of the COVID-19 disease in noise pollution. Although this analysis is out of the scope of the current work, readers should note that the impact of the COVID-19 lockdown period in noise levels and soundscapes has been analyzed in different cities, such as Barcelona [55] and Milan [56].

**Figure 3:**
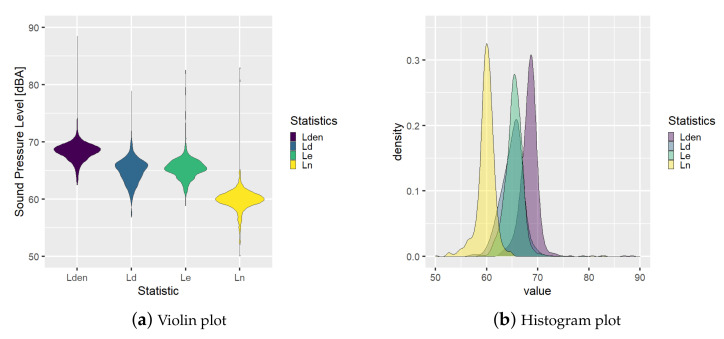
Statistics probability distribution analysis for Node $BCN_{1}$.

**Figure 4:**
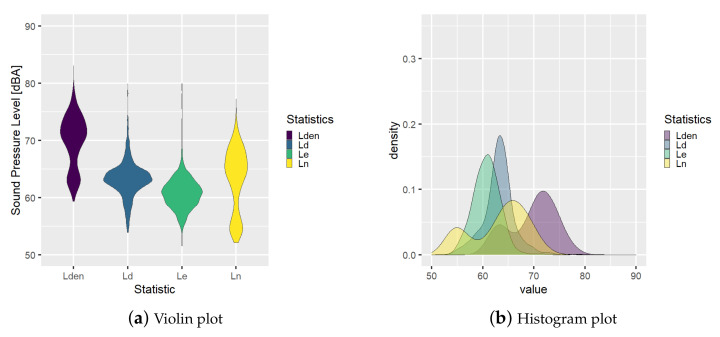
Statistics probability distribution analysis for Node $BCN_{27}$.

Moreover, a graphical analysis about the probability distribution of the statistics can be derived from these data. In the following Figure 3 and Figure 4, examples of distribution plots for $BCN_{1}$ and $BCN_{27}$, respectively, are shown finding different behaviors. Node $BCN_{1}$ has a mean sound pressure level during the night period lower than daily and evening, see Figure 3, but $BCN_{27}$ has a probability function for the night period with two modes with one peak with higher sound pressure level than the peak of the daily function, see Figure 4.

Additionally, the variability of the $BCN_{27}$ node’s statistics is higher than $BCN_{1}$ node’s ones. These statistics and the variability are going to be used in this work to model the node’s behavior.

### 3.3. Unsupervised Learning Modeling: K-Means

The goal of the modelling stage is to identify different behaviors in the acoustic nodes that can be correlated with the environmental impact and public health. As it was introduced in Section 2, clustering techniques learn patterns from data and group the elements in some clusters with the same behavior.

**Figure 5:**
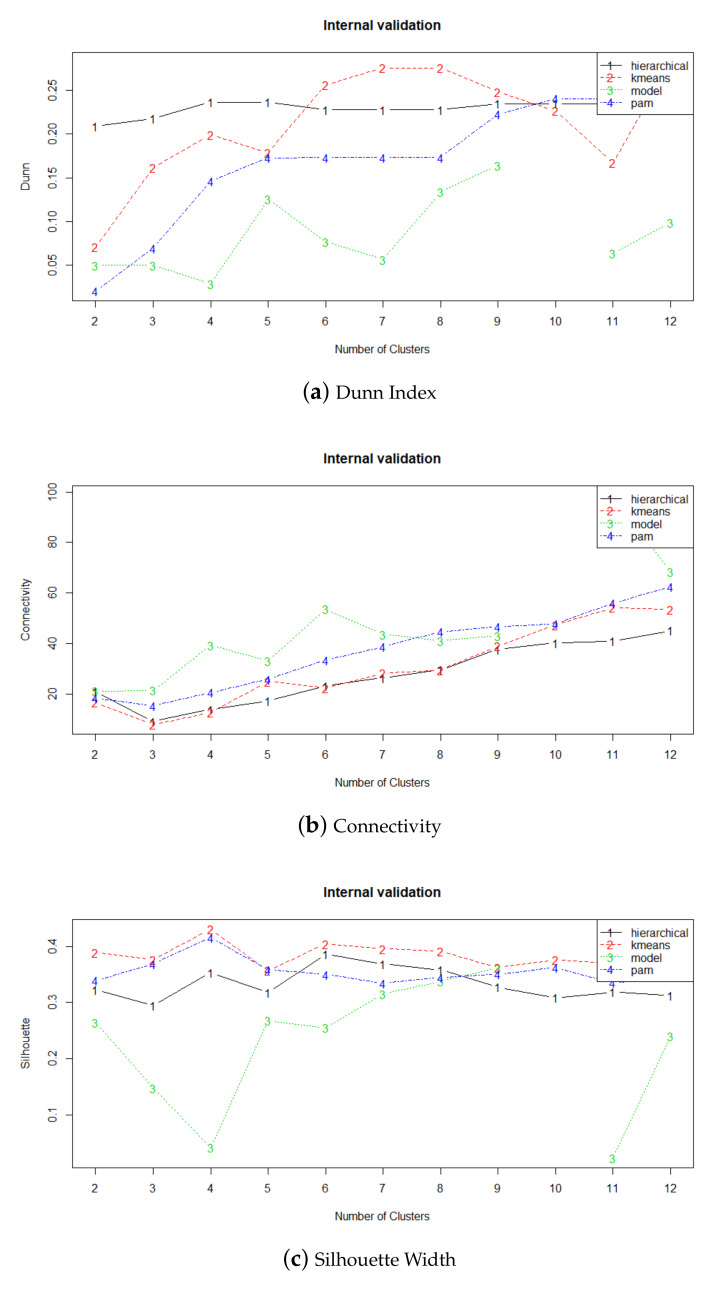
Validation measures for a given set of clustering algorithms and number of clusters.

In this research, several clustering algorithms were trained, including k-means clustering [57], hierarchical agglomeration [58], partitioning around medoids [59] and expectation maximization algorithm [60] using the following yearly acoustic indexes, $L_{\mathrm{day}}$, $L_{\mathrm{evening}}$, $L_{\mathrm{night}}$ and standard deviation of $L_{\mathrm{den}}$. A comparison of the results using Dunn Index [61], Connectivity [62] and Silhouette Width [63] concludes that k-means has the best performance for these data. Figure 5 shows that k-means maximizes Dunn Index and Silhouette Width and minimizes Connectivity.

The method considered in the following, called k-means clustering [57], is an unsupervised learning algorithm which groups the unlabeled data-set into different clusters, where *k* defines the number of predefined clusters that need to be created in the process.

This algorithm is iterative with two steps in each iteration t=1,2,…. In the first step, called the assignment step, each node is assigned to the nearest centroid using a distance. Thus, each node $N_{i}$ is assigned to the cluster centroid $C_{j}^{\left(t-1\right)}$ if $j={arg min}_{l=1}^{k}\left(d\left(N_{i},C_{l}^{\left(t-1\right)}\right)\right)$ where $d(N_{i},C_{l}^{\left(t-1\right)})$ represents the distance between node $N_{i}$ and cluster centroid $C_{l}^{\left(t-1\right)}$. In the second step, called the update step, the new centroids are calculated as the element-wise mean of the nodes assigned to each centroid using the following equation:

(4) $$C_{j}^{\left(t\right)}=\frac{\sum_{i\in A_{j}^{\left(t\right)}}N_{i}}{|A_{j}|},$$

where $A_{j}^{\left(t\right)}=\left\{i|N_{i}\mathrm{is}\;\mathrm{assigned}\;\mathrm{to}\;C_{j}^{\left(t-1\right)}\right\}$ is the set that includes the nodes assigned to the cluster centroid $C_{j}^{\left(t-1\right)}$ in the previous step.

The algorithm iterates this two steps until the centroids have stabilized, i.e., there is no change or it is residual in their values because the clustering has been successful or the defined number of iterations has been achieved. To initialise the algorithm, *k* random centroids $C_{j}^{\left(0\right)}$ are calculated for a chosen integer *k*. The euclidean distance was considered in this research, so for a node $N_{i}$ represented by their *m* components $N_{i}=\left(n_{i1},n_{i2},\ldots ,n_{im}\right)$ and *k* cluster centroids $C_{j}^{\left(t\right)}$ represented by their *m* components $C_{j}^{\left(t\right)}=\left(c_{j1}^{\left(t\right)},c_{j2}^{\left(t\right)},\ldots ,c_{jm}^{\left(t\right)}\right)$ the distance is defined by the following equation:

(5) $$d\left(N_{i},C_{j}^{\left(t\right)}\right)=\sqrt{\sum_{h=1}^{m}{\left(n_{ih}-c_{jh}^{\left(t\right)}\right)}^{2}}.$$

To avoid any variable to be dominant due to different measurement scales rather than relevance, the variables should be scaled to bring them down to a similar scale. Normalization, dividing the centered variables by their standard deviation ($\frac{X-\bar{X}}{\sigma_{X}}$ for every variable *X*), has been applied to data previous to the training of the k-means algorithm.

In this work, $L_{d1y}$, $L_{e1y}$ and $L_{n1y}$ indicators will be used as inputs to model the behavior of the nodes in different periods of the day, so the temporal variability during a day is taken into account. Moreover, yearly standard deviation of $L_{\mathrm{den}1d}$ to identify the variability of the nodes during a year, denoted as $sd_{1y}$($L_{\mathrm{den}1d}$). As a comparison will be performed with the SNM of the city, the selection of these variables as inputs is also based on Directive 2002/49/EC [4]. In particular, this Directive recommends as noise indicators $L_{\mathrm{den}1y}$ and $L_{n1y}$ for the preparation and revision of SNM, and where appropriate, $L_{d1y}$, and $L_{e1y}$, for road-traffic noise, rail-traffic noise, aircraft noise around airports and noise on industrial activity sites. Directive 2002/49/EC [4] also proposes that every five years, SNM showing the situation in the preceding calendar year should be carried out. However, this year should be a relevant year, as regards the emission of sound, and an average year, as regards the meteorological circumstances. In these terms, 2020 can not be considered as a relevant year due to COVID-19 pandemic lockdown. Therefore, 2019 is the most recent year with stable data.

**Figure 6:**
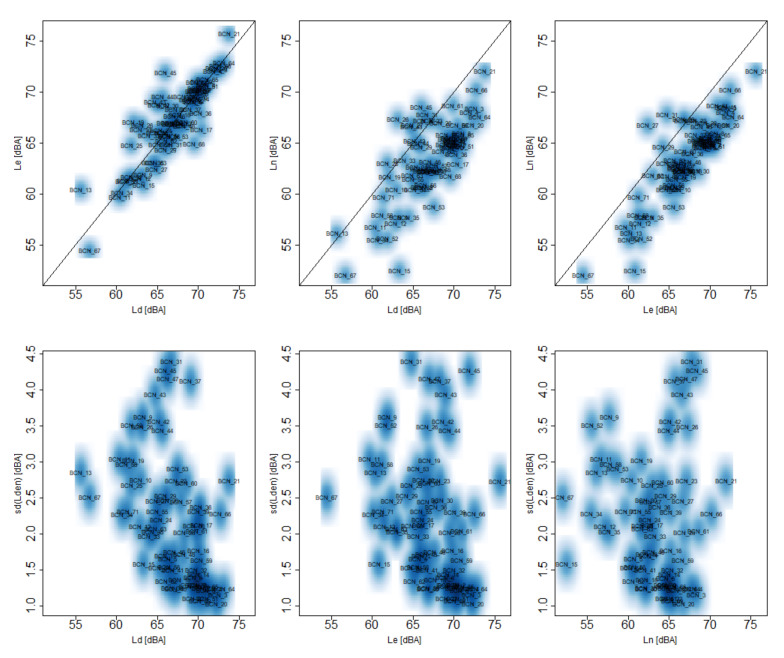
Scatter plot of the $L_{d2019}$, $L_{e2019}$, $L_{n2019}$, and $sd_{2019}$($L_{\mathrm{den}1d}$) metrics representing all the nodes. Black line represents identity line, i.e., equal value for both KPIs.

In order to show the relevance and the relation between these indicators, $L_{d2019}$, $L_{e2019}$, $L_{n2019}$ and $sd_{2019}$($L_{\mathrm{den}1d}$), a smoothed color density scatterplot representing all the nodes can be seen in Figure 6. The smoothed color density helps to identify dense zones that groups nodes with similar behavior. The first row of plots compares the sound pressure level statistics pairwise. The black line is the so-called identity line meaning that both statistics are equal. The nodes in the upper right part of each plot show high sound pressure level values that affects citizen well being. Moreover, it can be observed that there are nodes that $L_{n2019}$ is higher than $L_{d2019}$ or $L_{e2019}$, causing noise annoyance in the citizens. The second row of plots compares each sound pressure level statistic with the standard deviation of $L_{\mathrm{den}1d}$. In these plots, it can be identified different types of nodes: nodes with low sound pressure level and low standard deviation related with quite zones, nodes with high sound pressure level and low standard deviation related with a constant high noise pollution and nodes with high standard deviation that have some days with low sound pressure level and other days with high sound pressure level. A dense zone around the point $L_{d2019}$ = 70 dBA, $L_{e2019}$ = 70 dBA, $L_{n2019}$ = 65 dBA and $sd_{2019}$($L_{\mathrm{den}1d}$) = 1.2 dBA groups nodes with a constant noise pollution along both the day and the year.

**Figure 7:**
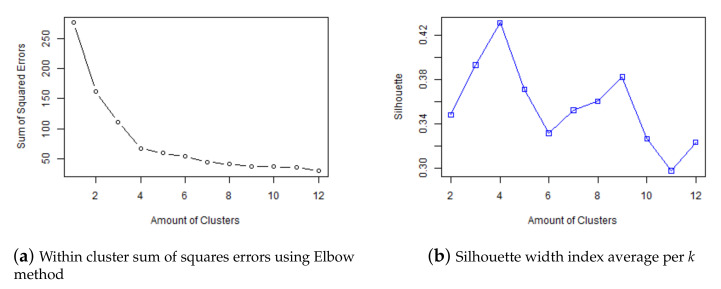
Amount of Cluster Selection Techniques.

An important fact of k-means algorithm is that the amount of clusters has to be fixed before the model is trained. To determinate the appropriate amount of clusters, k-means algorithm has been trained for k=1,…,12 and two amount of cluster selection techniques called *Elbow Method* [64] and *Silhouette* [63] have been considered. The selection of these techniques is based on the objective of the research, to find groups of nodes with the same behavior, so the focus is to evaluate the similarity of the nodes within the same cluster, independently of the rest of the clusters. Elbow and Silhouette are calculated on the relationship within the clusters. Figure 7a shows the within cluster sum of squares error for *k* clusters, with *k* from 1 to 12. The optimal *k* is the one related with the knee of the curve, i.e., the one that the increase in the number of clusters is not related with a high relative reduction in sum of squares error. In this case according to the Elbow Method, it is k=4 where the slope changes from −0.39 to −0.12. Figure 7b shows the average Silhouette width for *k* clusters, with *k* from 1 to 12 and the optimal *k* is the first maximum. In this case is *k* = 4, matching with the Elbow method estimation too.

As the k-means algorithm is randomly initialized, 100 experiments with random seeds were run to verify if a local or optimal solution is reached. In 96 of them, the solution presented in Table 3 in Section 4 was reached, obtaining the lowest sum of squares error for the same amount of clusters.

### 3.4. Software and Technology

The preparation, transformation, analysis and modelling of the data have been performed using the Statistical Programming Language R [65], combining a local environment using R version 3.5.1 with a cloud environment provided by RStudio Cloud using R version 4.0.3. The cloud environment has been used to parallelize some tasks. The following libraries have been involved in the tasks: stringr (Version 1.4.0), dplyr (Version 1.0.5), tidyr (Version 1.1.3), cluster (Version 2.1.1), ggplot2 (Version 3.3.3), hrbrthemes (Version 0.8.0), imputeTS (Version 3.2) and zoo (Version 1.8-9).

To ensure the reproducibility of the research, in every task that includes a random step, the seed using the R function set.seed() has been fixed. Due to changes in random numbers generation in R version 4.0.0, the way to generate them to be sure that the analysis will be reproducible in every R version has also been defined.

## 4. Results and Discussion

In this section, results obtained from applying the clustering technique, see Section 3.3 for details, to the collected data, see Section 3.2 for details, are analyzed and discussed. Firstly, the selection of the optimum amount of cluster *k* is reviewed, and a description of the selected clusters is detailed. Secondly, a spatial and a temporal analysis of the results are presented. Finally, a discussion about the results regarding the report from the SNM of Barcelona is presented.

### 4.1. Clustering Analysis

Although both selection methods agreed with *k* = 4 clusters, considering Silhouette metric *k* = 3, *k* = 4 and *k* = 9 clustering results have been analysed to compare the knowledge that can be extracted from them. For a given value of *k*, k-means algorithm groups the nodes in *k*-clusters. Then, the centroid is calculated for each cluster following Equation (4). These centroids help to identify the different behavioral patterns from an acoustic perspective. Centroids and features calculated for *k* = 3, 4 and 9 are shown in Table 3.

If *k* = 4 is chosen, the algorithm divides the nodes in four clusters related with high (cluster 1 or black), medium (cluster 2 or magenta) and low (cluster 4 or brown) ranges of sound pressure level values and another particular group (cluster 3 or cyan) with a singular behavior. On one hand, clusters 1, 2 and 4 have similar behavior, i.e., a comparable daily and evening sound pressure level values and a significantly lower night sound pressure level values. Moreover, the higher values, the lower variability that are shown in these three clusters. On the other hand, cluster 3 has almost the same daily and nightly sound pressure level values but higher evening sound pressure level values. Moreover, the nodes included in this cluster 3 show the highest variability during the year 2019. Regarding *k* = 3 case, the algorithm divide the nodes in three clusters, two of them related with high (cluster 1) and low (cluster 2) ranges of sound pressure level values and another particular group (cluster 3) with a similar behavior to the third one in *k* = 4 clustering. Finally, for a *k* = 9 value, the algorithm divides the nodes in nine clusters related with very high (cluster 5 and 9 with medium and low variance, respectively), high (cluster 1 and 2 with medium variance and cluster 6 with low variance), medium (cluster 7 and 8 with medium and low variance, respectively) and low (cluster 4) ranges of sound pressure level values. Although clustering with *k* = 9 identifies more behaviors than the others, some of the clusters have a small number of nodes ceasing to be statistically significant, for instance, cluster 5 has only two nodes. Moreover, proposing action plans for such a large number of clusters can be a complex and inefficient task from a practical point of view.

In conclusion, the three models identify the same particular group with different behavior (cluster 3 in all options) from the rest of nodes that are classify depending on their ranges of sound pressure level values. These results reinforce the selection of the optimal *k* = 4 value.

### 4.2. Description of k = 4 Clustering

**Figure 8:**
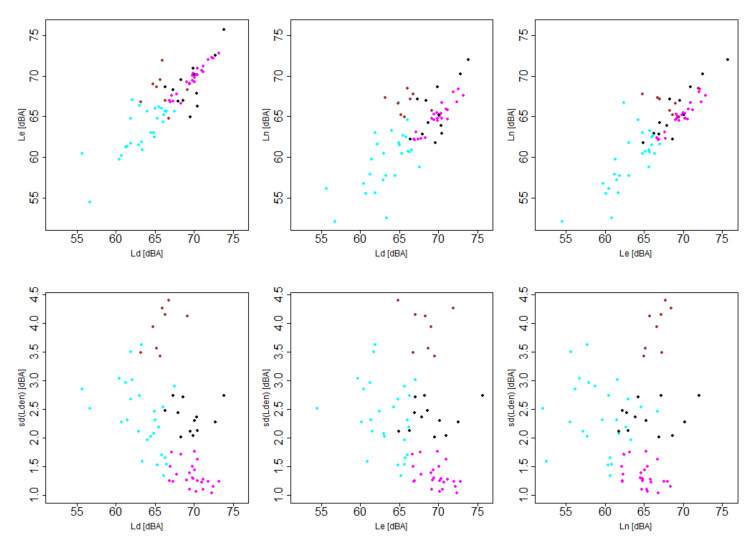
Nodes assignment to each cluster based on centroids for *k* = 4. Color legend: cluster 1 (black), cluster 2 (magenta), cluster 3 (cyan) and cluster 4 (brown).

Once the quantity of clusters is fixed to *k* = 4, every node is assigned to a cluster depending on its distance to the centroids, as it is graphed in Figure 8.

Regarding the different range of values, cluster 4 belongs to the range with the lowest sound pressure level values and cluster 2 is in the intermediate values range, as can be seen in the top row graphs of Figure 8. Furthermore, clusters 1 and 3 contain the nodes with the highest sound pressure level values. Bottom row graphs of Figure 8 show the relation of the clusters with the variability. In one hand, cluster 3 presents high variability in the three periods of the day that can represents an acoustic environment with discontinuous and impulsive sound sources. In the other hand, cluster 1 presents low variability so the citizen are exposed to an acoustic environment where constant and stationary sound sources are predominant.

### 4.3. Geographical Analysis of the Clusters

**Figure 9:**
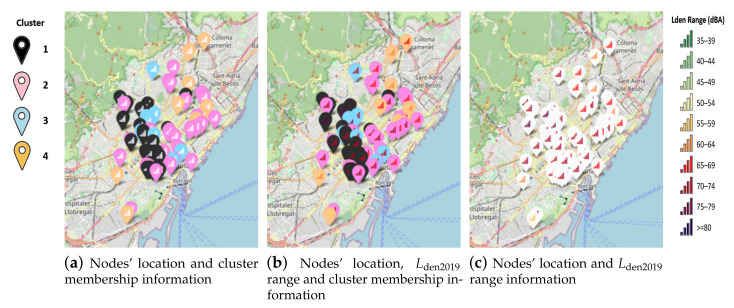
Maps developed for the geographical analysis with combined spatial, sound level and cluster information.

A SNM is a set of maps that serve to globally assess the population’s exposure to noise produced by different noise sources in a given area, and to serve as the basis for the development of action plans in a city. Moreover, they have to be updated periodically, at least every 5 years. Therefore, it can be helpful to figure out the geographic relationship between the acoustics nodes, to identify areas of the city that are related with the clusters. Taking advantage of the performed *k* = 4 clustering, it is possible to combine the results with the spatial information to perform a geographical analysis of the city’s sound environments. Figure 9 shows three maps where the location icons represents the node’s location. If the location icon is colored, the color represents the assigned cluster. Inside the icon, there is a plot symbol that shows, if colored, the equivalent sound pressure level $L_{\mathrm{den}2019}$ according to ISO 1999 [53].

Looking at the maps, geographic patterns appear with concentrations of nodes of the same cluster in areas of the city. Nodes of the cluster 1, those with the highest sound pressure level values, are located in the southwestern section of the city, while the northeastern section is related with lower sound pressure level values. More details can be obtained if the location of nodes belonging to cluster 1 is consulted. These nodes are related with locations near wide streets with high volume of vehicular traffic in south and west of the city such as *Avinguda Paralell* (nodes $BCN_{2}$, $BCN_{3}$, $BCN_{4}$ and $BCN_{32}$), *Avinguda Diagonal* (nodes $BCN_{8}$, $BCN_{14}$, $BCN_{16}$ and $BCN_{39}$) *Travessera de Dalt* (nodes $BCN_{20}$, $BCN_{21}$, $BCN_{64}$, $BCN_{65}$ and $BCN_{66}$) which are the natural entrances to Barcelona city. Regarding cluster 3, it has been found that the location of its nodes is related with evening and nightly leisure, zones with some pubs such as *La Ribera* (node $BCN_{26}$), *Carrers del Escudellers* (node $BCN_{23}$), entertainment zones such as *Gracia District* (nodes $BCN_{43}$, $BCN_{44}$, $BCN_{45}$ and $BCN_{47}$) or shopping streets such as *Passeig de Gracia* (node $BCN_{37}$).

The maps included in Figure 1 and Figure 9 are available in an interactive discovery version, developed in python [66], accessible by this github repository link [67] clicking on *Open in Colab* button (accessed on 16 May 2021).

### 4.4. Temporal Analysis of the Clusters

In a big city like Barcelona, acoustic environments may change over time due to sound sources mobility in space and variability in time and amplitude. There may be several reasons for these changes, among them the following are worth mentioning: modifications in the mobility of the citizens, effects derived by the SNM’s action plans to improve the acoustic quality of the city, tourism and leisure places reallocation or special situations such as a lockdown derived by a pandemic situation. So, it is important to monitor the evolution of the statistics and the implications in the clusters composition.

As presented in Section 3.3, the k-means method was trained with a one year data, also called window, in particular 2019, allowing to identify the node’s statistics and the cluster to which belongs. Once the clusters have been identified, it is possible to investigate behavioral changes of nodes over time using a sliding window. This monitoring technique can be related with a long-term noise pollution strategy, if a one-year sliding window is considered to have enough previous information. The data-set includes data from January 2018 until December 2020, thus the node’s cluster to which it belongs is calculated from 31 December 2018 until 31 December 2020.

**Figure 10:**
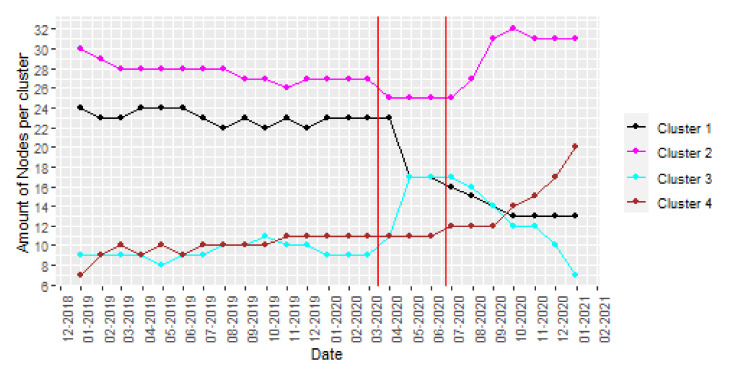
*k* = 4 clusters assignment (yearly sliding window).Note that Spanish lockdown corresponds with the period between red lines.

Firstly, a study of the monthly evolution with a yearly sliding window of the amount of nodes per cluster is represented in Figure 10. Sound sources may have seasonality due to external effects, such as tourism or work-periods, that change during seasons of the year. Therefore, a one-year window is appropriate to identify trend, cycle patterns or special events because it is not affected by seasonality.

The graphs in Figure 10 show that, before lockdown was established in March 2020, the cluster distribution is stable. Note a small reduction in clusters 1 and 2, those in the ranges with higher sound pressure level values, indicating that noise pollution was decreasing in Barcelona. During the lockdown period, a noticeable increase in the amount of nodes belonging to the cluster 3 appears as expected, as this cluster is linked with higher variability. As the size of the sliding window is one year, this monitoring analysis helps smart cities to identify the tendency of the acoustic environments and the long-term effects of action plans.

**Figure 11:**
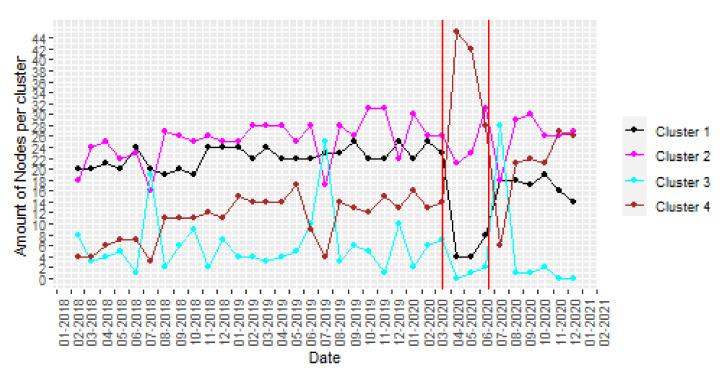
*k* = 4 clusters assignment (monthly sliding window). Note that Spanish lockdown corresponds with the period between red lines.

Reducing the size of the sliding window to one month previous to the date, short-term changes and seasonality can be observed. Then, a study of the monthly evolution with a monthly sliding window of the amount of nodes per cluster is represented in Figure 11.

It can be observed in Figure 11 that there was a seasonal variation in July of every year, causing an increase in the amount of nodes belonging to cluster 3. When lockdown was established in March 2020, there was a significant change in the noise pollution, increasing the nodes belonging to cluster 4 that is related with quiet areas. After the state of alarm was over by the end of June, the clusters’ distribution became similar to the previous pandemic situation except for a small reduction in clusters 1 and 3, which is related with a lower noise pollution.

As a conclusion, once the unsupervised algorithm is trained, this temporal analysis with different sliding window sizes can help city managers to properly monitor acoustic areas and their noise pollution according to their objectives. Moreover, new nodes could be included in this monitoring model allowing to estimate the area to which they would be assigned and also to compare with nodes in other zones or cities.

### 4.5. Discussion Regarding the Machine Learning Model Results and the City SNM Report

The SNM of Barcelona city [14,68], which last release is from 2017, is divided into three different maps and related reports that graphically describe the exposure of the citizens to the sound sources in different areas following the recommendations of the Directive 2002/49/EC [4]. The noise map, *Mapa de Soroll* in Catalan, shows the sound pressure levels using isophonic curves coming from different sources and in different time periods. The capacity map, *Mapa de Capacitat* in Catalan, classifies the city in zones of different acoustic sensitivity, determining the maximum limits of noise permitted by regulations. Finally, the sites that exceed the permitted levels are included in the overcoming map, *Mapa de Superació* in Catalan.

Examining the places that were identified in the overcoming map, a comparison has been performed with the results obtained with the proposed machine learning method. Table 4 contains an overview of this comparison. In the first two columns, a list of the zones that are highlighted in SNM report for the overcoming map and their classification in different periods of the day are shown. In the rest of the columns, a count of the nodes per cluster corresponding to each zone has been performed including the total amount of nodes per zone in the last column. In general, Table 4 shows that cluster 1 is mainly related with day and evening periods in the overcoming map, while cluster 3 is mainly related with night period.

The first four rows of Table 4 present information regarding places where day and evening periods have a high level of noise exposure. The following are included in the overcoming map: *Sarrià - Sant Gervasi*, corresponding with node $BCN_{18}$ from cluster 2 and nodes $BCN_{22}$, $BCN_{50}$ from cluster 1, *Avinguda Diagonal* corresponding with nodes $BCN_{14}$, $BCN_{16}$ and $BCN_{39}$ from cluster 1 and $BCN_{17}$ and $BCN_{36}$ from cluster 2, *Ronda General Mitre* corresponding with nodes $BCN_{20}$, $BCN_{21}$, $BCN_{64}$, $BCN_{65}$ and $BCN_{66}$ from cluster 1 and *Carrer Balmes* corresponding with nodes $BCN_{6}$, $BCN_{7}$ and $BCN_{8}$ from cluster 1. In summary, cluster 1 has 13 of its 23 nodes located in zones that are included in the overcoming map. The other five nodes, except node $BCN_{51}$ that is far from these zones, are near to the previous places. It is important to mention that the remaining four nodes of cluster 1 are related with *Avinguda Parallel* which was not included in the SNM report, but the machine learning method has identified them. Therefore, it is recommended to include them in the next release of this overcoming map.

The last two rows of Table 4 present information regarding places where the night period has a high level of noise exposure, and the following are included in the overcoming map: *Sarrià-Sant Gervasi*, corresponding with node $BCN_{18}$ from cluster 2 and nodes $BCN_{22}$ and $BCN_{50}$ from cluster 1, *Gràcia*, corresponding with nodes $BCN_{43}$, $BCN_{44}$, $BCN_{45}$ and $BCN_{47}$ from cluster 3 and *Ciutat Vella*, corresponding with nodes $BCN_{10}$, $BCN_{24}$, $BCN_{27}$ and $BCN_{53}$ from cluster 2 and nodes $BCN_{23}$ and $BCN_{26}$ from cluster 3.

Barcelona city council has also defined in the SNM report two Special Regimen Acoustic Zones (SRAZ), which are subzones of the previously commented, related with nightly entertainment activities. These two zones are *Vila de Gràcia*, corresponding with nodes $BCN_{43}$, $BCN_{44}$, $BCN_{46}$ and $BCN_{47}$ from cluster 3 and *Barri Gótic i Rambla del Raval*, corresponding with node $BCN_{24}$ and $BCN_{27}$ from cluster 2 and nodes $BCN_{23}$ from cluster 3. In summary, cluster 3 has 9 elements, 5 of them are included in the SRAZ and can be observed in Figure 9. Other nodes from cluster 3 but not included in the SRAZ are near them, except node $BCN_{42}$ which is isolated from the rest. As a result, the proposed unsupervised learning technique can help to identify new locations with certain acoustic conditions.

## 5. Conclusions

Urban acoustic environments should be continuously monitored in large cities, because of the fact that sound sources affect the well-being and quality of life of citizens. In recent years, wireless acoustic sensor networks have been deployed in cities to capture information about the sound environment over long periods of time and at many locations. This network of sensors generates huge amount of data that can not be simply processed but a machine learning algorithm can be applied in order to obtain data insights, predictions and relevant information from the data.

This paper has presented the analysis of urban acoustic environments applying unsupervised machine learning techniques, specifically k-means method, to identify and classify different acoustic profiles of the city using yearly averaged sound pressure level indicators as input of the clustering approach. It has been shown that the k-means method can find out relationships between input variables and group the node locations according to their similarity. This technique does not need labeled input and output data to train the model and automatically create clusters of nodes that share an acoustic behavior. To explore the suitability of this technique, sound pressure level values acquired by 70 acoustic nodes during a three year campaign in the city of Barcelona have been used. After the data-set was prepared, different acoustic indicators, $L_{\mathrm{den}}$, $L_{\mathrm{day}}$, $L_{\mathrm{evening}}$, $L_{\mathrm{night}}$ and some statistics have been calculated to train several algorithms with clean and adequate feature inputs.

The modelling phase has been carried out using yearly average indicators from data of 2019, because it has shown to be a reference year regarding Directive 2002/49/EC. The optimum amount of clusters has been chosen using Elbow and Silhouette methods, resulting in *k* = 4. However, clustering with *k* = 3 and *k* = 9 have been also analyzed to compare the knowledge that can be extracted from them. In general, two different behaviors have been detected. One type where clusters have higher sound pressure level values during day and evening periods than during night period, and other type where sound pressure level values are higher during evening period than during day and night periods. Moreover, as the average sound pressure level of the cluster increases the variability of the values decreases.

After the model is developed, acoustic nodes have been assigned to created clusters to perform both spatial and temporal analysis of the results. The geographical analysis allows to identify areas of the city that are related with the different clusters and detect relationship between the acoustics nodes. Applying different sliding window sizes, behavioral changes over time have been investigated. With a size of one year, the tendency of the acoustic environments and the long-term effects of action plans have been analyzed. Reducing the size of the sliding window to one month, short-term changes and seasonality effects have been studied. Finally, a comparison between the results obtained by the machine learning model and the last strategy noise mapping report from the city has been performed. Most of the locations appearing on the overcoming map have been found with the developed k-means model. In addition, an area has been discovered that should be considered within the overcoming map in the next revision of the map. Moreover, the developed model can be applied regularly to detect nodes with similar behavior to previously identified clusters and to follow the temporal evolution of the clusters.

## Figures and Tables

**Table 1:** Example of data completeness analysis for several acoustic nodes.

Node ID	First Day	Days with Records	Total Records	Valid	Null	% Null
$BCN_{1}$	9 July 2018	853	1,228,320	1,208,463	19,857	1.62%
$BCN_{2}$	1 January 2018	1031	1,484,640	1,449,202	35,438	2.39%
$BCN_{3}$	1 January 2018	1037	1,493,280	1,468,077	25,203	1.69%
$BCN_{4}$	1 January 2018	1047	1,507,680	1,485,358	22,322	1.48%
$BCN_{5}$	10 October 2018	762	1,097,280	1,074,836	22,444	2.05%
$BCN_{6}$	1 January 2018	1043	1,501,920	1,477,480	24,440	1.63%
$BCN_{7}$	1 January 2018	1044	1,503,360	1,477,990	25,370	1.69%
$BCN_{8}$	1 January 2018	980	1,411,200	1,384,799	26,401	1.87%
$BCN_{9}$	1 January 2018	1042	1,500,480	1,476,821	23,659	1.58%
$BCN_{10}$	3 September 2018	813	1,170,720	1,162,474	8246	0.70%

**Table 2:** Example of summary statistics for node $BCN_{1}$.

Node ID	Date	$L_{d1d}$	$L_{e1d}$	$L_{n1d}$	$L_{\mathrm{den}1d}$	$L_{011d}$	$L_{101d}$	$L_{501d}$	$L_{901d}$	$L_{991d}$
$BCN_{1}$	1 August 2018	64.48	64.64	59.09	67.51	69.36	66.10	62.60	52.49	41.44
$BCN_{1}$	2 August 2018	64.74	64.89	59.10	67.65	70.20	66.30	62.70	53.79	40.94
$BCN_{1}$	3 August 2018	64.66	64.72	59.35	67.70	70.06	66.10	62.70	54.09	44.56
$BCN_{1}$	4 August 2018	62.09	62.95	58.15	66.05	67.20	63.70	60.20	53.08	42.34
$BCN_{1}$	5 August 2018	61.56	62.09	58.21	65.77	66.36	62.90	59.10	51.90	41.30
$BCN_{1}$	6 August 2018	63.60	64.28	59.22	67.28	68.56	65.40	62.10	52.00	42.94
$BCN_{1}$	7 August 2018	63.84	63.72	59.18	67.17	69.62	65.40	62.10	51.80	41.24
$BCN_{1}$	8 August 2018	64.91	64.00	59.26	67.55	69.70	65.91	62.30	52.19	43.38
$BCN_{1}$	9 August 2018	65.21	63.82	59.50	67.71	70.30	66.90	62.70	53.70	42.20
$BCN_{1}$	10 August 2018	64.05	65.19	60.21	68.14	69.68	66.00	62.50	53.90	41.56
$BCN_{1}$	11 August 2018	63.17	63.42	58.97	66.84	70.56	64.60	60.70	52.80	43.54
$BCN_{1}$	12 August 2018	60.48	62.86	57.70	65.49	68.00	62.80	58.80	50.79	39.64
$BCN_{1}$	13August 2018	64.72	64.19	59.32	67.57	71.94	65.90	61.90	51.70	41.16
$BCN_{1}$	14 August 2018	64.28	64.38	58.92	67.31	70.46	65.90	62.20	52.09	41.34
$BCN_{1}$	15 August 2018	60.83	62.34	59.40	66.45	67.10	63.50	59.70	52.29	41.34

**Table 3:** Size and centroids of clusters for *k*=3, 4 and 9 using data collected during 2019.

Cluster	$L_{d2019}$	$L_{e2019}$	$L_{n2019}$	${\mathrm{sd}}_{2019}$($L_{\mathrm{den}1d}$)	Size
k-means with 3 clusters					
1	69.85	69.50	65.12	1.64	35
2	63.36	63.27	59.54	2.36	26
3	66.05	68.25	66.71	3.78	9
k-means with 4 clusters					
1	70.74	70.79	66.39	1.50	23
2	66.40	66.04	62.28	2.06	27
3	66.05	68.25	66.71	3.78	9
4	61.11	60.57	56.24	2.61	11
k-means with 9 clusters					
1	68.92	69.72	67.00	2.27	4
2	68.69	66.75	62.37	2.44	7
3	65.89	68.25	66.65	3.92	8
4	60.78	60.33	56.09	2.67	10
5	73.30	74.11	71.11	2.50	2
6	70.16	70.00	65.23	1.32	14
7	64.19	64.89	62.33	2.40	10
8	66.78	66.37	61.76	1.50	11
9	72.46	72.34	67.69	1.16	4

**Table 4:** Overcoming map zones and clusters summary. Note that percentage (X%) is calculate over each zone. Note that d, e and n in brackets correspond to day, evening and night period, respectively.

Zone	SNM Class	Cluster 1	Cluster 2	Cluster 3	Cluster 4	Total Zone
*Sarrià-Sant Gervasi*	Overcoming Map (d, e and n)	2 (67%)	1 (33%)	0 (0%)	0 (0%)	3
*Avinguda Diagonal*	Overcoming Map (d and e)	3 (60%)	2 (40%)	0 (0%)	0 (0%)	5
*Ronda General Mitre*	Overcoming Map (d and e)	5 (100%)	0 (0%)	0 (0%)	0 (0%)	5
*Carrer Balmes*	Overcoming Map (d and e)	3 (100%)	0 (0%)	0 (0%)	0 (0%)	3
*Avinguda Parallel*	No included in SNM	4 (80%)	1 (20%)	0 (0%)	0 (0%)	5
*Gràcia*	Overcoming Map (n)	0 (0%)	0 (0%)	4 (100%)	0 (0%)	4
*Ciutat Vella*	Overcoming Map (n)	0 (0%)	4 (67%)	2 (33%)	0 (0%)	6
*Others*	—–	6 (15%)	19 (49%)	3 (8%)	11 (28%)	39
Total	—–	23	27	9	11	N = 70

## Data Availability

The data-set used in this research has been provided by the city council of Barcelona, but authors of this research do not have permission to publish it. However the maps included in Figure 1 and Figure 9 are available in an interactive discovery version, in github repository link [67], accessed on 16 May 2021.
